# A New Method of Effectually Remedying the Defect of Hare-lip

**Published:** 1870-12

**Authors:** 


A New Method of Effectually Remedying the Defect of Hare-lip.—Dr. Wm. Stokes, Jr., Surgeon to the Richmond Surgical Hospital (*Dublin Quarterly Journal*), presents, with plates, an ingenious operation for remedying the congenital malformation called hare-lip, which prevents the formation of a notch; also gets rid of the chance of that defect subsequently occurring. By his operation he gets rid of all the defects of other procedures, in which, in part or entirely, the principle of the utilization of parings has been adopted. For—

*First.* No subsequent curtailment of the projection at the lower extremity of the cleft is necessary.

*Second.* The procedure is applicable to all forms and varieties of hare-lip.

*Third.* There is no chance of portions of the soft tissues perishing from any twisting of them.

*Fourth.* There can be no subsequent puckering.

*Fifth.* There can be no subsequent notch, as previously mentioned.

*Sixth.* The possibility of any vertical sulcus or groove in the line of the cicatrix is also prevented, which is the case after Sédillot's, Malgaigne's, Samuel Smith's, and other operations.

(The reader is referred to the author's extended remarks upon the operation, accompanied with cuts, which are so interesting and connected that we should do injustice to Dr. Stokes by making extracts.)

As regards the time which is best for the performance of operations remedying this defect, surgeons are much divided in opinion; some eminent operators maintaining that where it is possible all such operations should be performed before dentition, while others hold that better results are obtained in all cases where the procedure has been deferred until a later period. His experience leads him to adopt the views of the latter class, and his reasons are as follows: *First.* That the structures at the period of life previous to dentition are so weak, soft, and pulpy, they are ill able to bear the continuous pressure of the needles and sutures, and that consequently they are specially liable to the occurrence of ulceration and the subsequent formation of transverse cicatrices. *Second.* The hemorrhage, which, even though it be extremely slight in amount, is, in very young subjects, fraught with some danger. *Third.* There is much difficulty in executing any of the plans that have been devised for the avoidance of the defects which some times subsequently occur. In support of his opinion he has the high authority of his colleague, Mr. Adams, and the late Dr. Samuel Maclean, who went so far as to assert that, if possible, in all cases operation should be deferred until after second dentition.—*Med. Record.*

